# Therapeutic hypoxia for mitochondrial disease via enhancement of hemoglobin affinity and inhibition of HIF-2**α**

**DOI:** 10.1172/JCI185569

**Published:** 2024-12-02

**Authors:** Hong Wang, Maria Miranda, Eizo Marutani, Paul Lichtenegger, Gregory R. Wojtkiewicz, Fumito Ichinose, Vamsi K. Mootha

**Affiliations:** 1 Howard Hughes Medical Institute and Department of Molecular Biology, Massachusetts General Hospital, Boston, Massachusetts, USA.; 2 Broad Institute, Cambridge, Massachusetts, USA.; 3 Department of Systems Biology, Harvard Medical School, Boston, Massachusetts, USA.; 4 Anesthesia Center for Critical Care Research and; 5 Center for Systems Biology, Massachusetts General Hospital, Boston, Massachusetts, USA.

**Keywords:** Genetics, Hypoxia

**To the Editor:** Preclinical studies have demonstrated the therapeutic potential of hypoxia for treating mitochondrial disorders. In the *Ndufs4*-KO mouse model of Leigh syndrome and the sh*Fxn* model of Friedreich’s ataxia, continuous breathing of 11% oxygen can prevent and reverse neurological disease, while 55% oxygen accelerates disease (1–3). Multiple mechanisms likely underlie the benefits of hypoxia, including attenuation of oxygen toxicity from brain hyperoxia, restoration of Fe-S clusters, and normalization of oxygen sensing. Alternative means of reducing oxygen delivery, including sublethal carbon monoxide and severe anemia, also reverse brain disease in *Ndufs4*-KO mice (4). Intermittent regimens of inhaled hypoxic air — 16 hours of 11% and 8 hours of 21% — have proven ineffective (3) — probably due to a compensatory, HIF-2α–dependent increase in hemoglobin (Hb) that, combined with periods of 21% oxygen, may be detrimental (5). Collectively, these studies highlight the potential of hypoxia therapy but also underscore the need for more practical modalities that are safe and effective.

Here, we report the development of a “hypoxia-in-a-pill” regimen that leverages two approved drugs (Figure 1A). To reduce oxygen delivery, we utilized GBT440, an allosteric activator of oxygen affinity recently approved for sickle cell anemia (6). The erythroid response to the resulting tissue hypoxia would limit the durability of this single agent. To counter this response, we combined PT2399, a member of a new class of HIF-2α inhibitors approved for renal cell cancer. We began by treating WT mice with each drug, individually or in combination, by oral gavage five days/week. After three weeks, GBT440 increased Hb from 15.54 g/dL to 17.65 g/dL (*n* = 8; *P* = 0.003), while PT2399 decreased it to 13.58 g/dL (*n* = 5; *P* = 0.015). The combination, however, led to Hb levels comparable to those with vehicle treatment (14.94 g/dL; *n* = 7; *P* = 0.6), indicating that GBT440 led to a HIF-2α–driven erythroid response (Figure 1B). Using an optical probe, we found that GBT440 increased PbO_2_ from 23.78 mmHg to 32.26 mmHg (*n* = 5; *P* = 0.001), probably because of increased Hb, whereas PT2399 decreased it to 17.55 mmHg (*n* = 6; *P* = 0.009). The combination decreased PbO_2_ to 15.23 mmHg (*n* = 6; *P* = 0.0006) (Figure 1C), comparable to what we previously achieved in mice breathing 11% FIO_2_ (see Figure 2D in ref. 4), and was well tolerated for more than two months (Figure 1D).

Having established that our drug combination could safely achieve tissue hypoxia, we tested its efficacy in *Ndufs4*-KO mice, beginning treatment on day 30. These mice are born healthy, show neurological defects on day ~35, and ultimately succumb at ~2 months of age to a fatal neurodegenerative disease resembling human Leigh syndrome. Open-field testing in *Ndufs4*-KO mice with advanced disease showed that the combination increased distance traveled from 4.8 m to 11.8 m (*P* = 0.04 Figure 1E). Brain MRI revealed characteristic T2-intense, Leigh-like lesions and/or hemorrhages in vestibular or cerebellar nuclei that were attenuated or even absent with the combination (Figure 1F). Although neither drug individually affected lifespan, the combination extended median lifespan by 30% from approximately 70 to 98 days and maximum lifespan from 80 to 144 days (*P <* 0.0001) (Figure 1G).

Our results provide preclinical proof of concept that simultaneously enhancing Hb oxygen affinity while antagonizing HIF-2α can mimic the effects of continuous hypoxic breathing for therapeutic benefit. The regimen did not confer as impressive a lifespan rescue as continuous breathing of 11% oxygen, probably because GBT440 has a short half-life (6), and for practical reasons, we treated the mice five weekdays per week. Future studies in humans are required to evaluate the safety of this combination, given that hypoxia can be associated with acute and long-term side effects. Such safety studies could pave the path for first-in-human “hypoxia-in-a-pill” trials in patients with mitochondrial disease.

## Supplementary Material

Supplemental data

Supporting data values

## Figures and Tables

**Figure 1:**
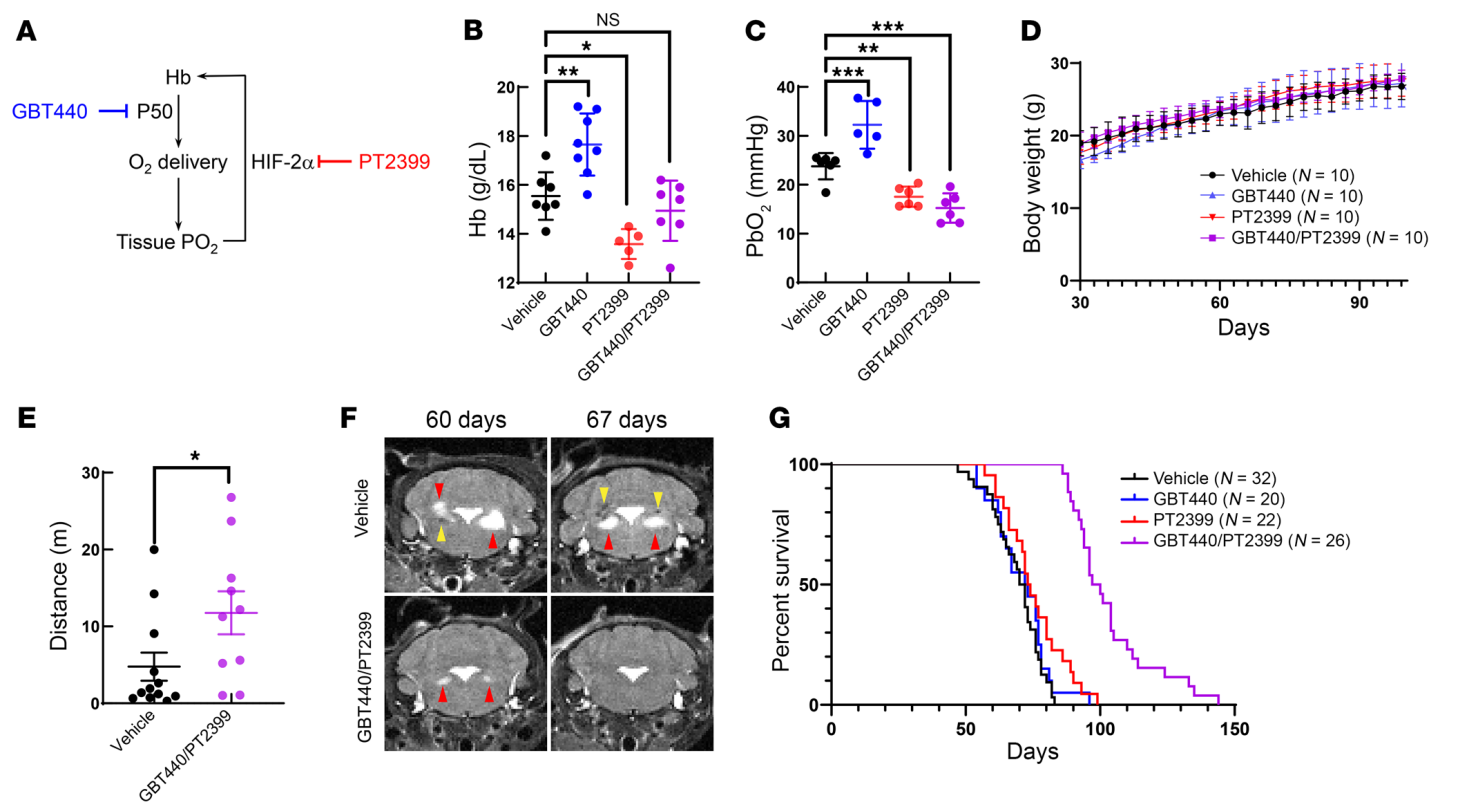
Small-molecule drug combination for therapeutic hypoxia. (**A**) Schematic overview of the “hypoxia-in-a-pill” regimen. GBT440 is an orally available activator of Hb affinity that, in theory, can reduce tissue oxygen delivery. In response to hypoxia, the body will mount a compensatory, erythroid response driven by HIF-2α, which is inhibited by PT2399. (**B**) Hb and (**C**) brain PbO_2_ measurements in 8-week-old WT mice treated with vehicle, GBT440, PT2399 or the combination for 3 weeks. (**D**) Body weight of WT mice treated with the indicated drugs. (**E**) Distance traveled in 15 minutes on an open-field test of *Ndufs4*-KO mice treated with vehicle or the GBT440/PT2399 combination. (**F**) Representative T2-MRI of *Ndufs4*-KO mice treated with vehicle or the combination at 60 and 67 days of age. Red arrowheads, Leigh-like lesion; yellow arrowheads, hemorrhage. (**G**) Survival of *Ndufs4*-KO mice treated with vehicle, GBT440, PT2399, or a combination. Bar plots show the mean ± SD. *n* = group size. *P < 0.05, **P < 0.01, ***P < 0.001; 1-way ANOVA with Dunnett’s test for multiple comparisons with vehicle; *t* test for single comparisons of GBT440/PT2399 versus vehicle; log-rank test for survival of drug-versus vehicle–treated mice.

## Version 1 12/02/2024

Electronic publication
