# Supplementary material for: Therapeutic hypoxia for mitochondrial disease via enhancement of hemoglobin affinity and inhibition of HIF-2α
Source: J Clin Invest. 2024 Dec 2;134(23):e185569. doi: 10.1172/JCI185569 (PMC11601899; doi:10.1172/JCI185569)
Supplement: Supplemental data [file jci-134-185569-s090.pdf]

SUPPLEMENTARY MATERIALS AND METHODS

Mouse strains and husbandry

Ndufs4 KO mice were generously provided by the Palmiter laboratory (University of Washington) (*1*, *2*). Pups were genotyped and weaned at 21-28 d of age. All cages were provided with food and water *ad libitum* and supporting Napa gel as needed. Mice were maintained in a standard 12h light-dark cycle at a temperature between 20-25°C and humidity between 30-70%. Homozygous WT and heterozygous KO were used as littermate controls for *Ndufs4* KO mice as they appear identical in all assays performed by us and others. Natural death or 20% body weight loss (euthanasia criteria) were used to generate survival curves. WT C57Bl6/J mice were purchased from The Jackson Laboratory and used for PK/PD studies.

Drug Administration

GBT440 and PT2399 were purchased from MedChemExpress. *Ndufs4* KO and WT mice were administered drugs by oral gavage five days per week: in the morning with 100 mg/kg of the HIF-2α inhibitor PT2399 and in the afternoon with 200 mg/kg of the left-shifter GBT-440 for the duration of their lifespan starting at 30 days of age. In murine models, PT2399 blocks the erythroid response to continuous, hypoxic breathing (*3*). Vehicle-treated mice were administered the vehicle for PT2399 (10% Ethanol, 30% PEG400, 0.3% Methyl cellulose, 0.3% Tween 80) in the morning and the vehicle for GBT-440 (5% DMSO, 0.25% SDS, 0.25% Methyl Cellulose) in the afternoon.

Brain PO_2 measurements

Wild-type C57BL/6J mice were anesthetized with isoflurane (induction at 2-4%, maintenance at 1-1.5%), intubated, and mechanically ventilated with a tidal volume of 8 ml/kg, a respiratory rate of 110 breaths per minute and an inspired fraction of oxygen (FiO_2) of 21%. Mice were placed in a prone position and the head was stabilized using a stereotaxic frame (ASI Instruments, MI). Rectal temperature was maintained at 37°C using a heating pad. After incision and dissection of the skin, an opening in the skull was performed using a micro-drill (MD-1200, Braintree Scientific, MA) and an optical PO_2 probe (OxyLab BF/OT/E, Oxford Optronix, Abingdon, UK) was inserted in the vestibular nuclei at the coordinates of ML = –1.25 mm, AP = –6.00 mm, and DV = –3.90 mm from the bregma. During the brain PO_2 measurement, the depth of anesthesia was reduced by lowering the Isoflurane concentration to 1% to minimize the impact of anesthesia on the brain PO_2.

Hemoglobin and hematocrit measurements

Eighty microliters of blood were collected by tail snip into a heparinized capillary. Hemoglobin concentration, hematocrit, and the saturation of hemoglobin with oxygen (O_2Hb) were measured using a blood gas analyzer (ABL800 FLEX, Radiometer, Copenhagen, Denmark).

Open-Field Test

The apparatus consisted of four open-field boxes measuring 50x50 cm. Each box was surrounded by opaque black walls, and the whole apparatus was shielded from outside influences by walls with a height of 1.5 meters. After an acclimation period of 60

minutes inside the procedure room, one mouse was placed into the center of an open-field box. Up to four mice were tested in the apparatus simultaneously. The animals were allowed to explore the field freely for 15 minutes while being recorded by the ANY-maze software with an overhead camera (v7.3, Stoelting Co., Wood Dale, IL). The position and movement of the animals were automatically tracked and analyzed by the ANY-maze software. The experimenters were blind to the treatment groups and, after starting the recording, left the room for the duration of the test. Before and after each test run, 70% ethanol was used to clean the apparatus.

MRI

Mice were continuously anesthetized with 0.5–1.0% isoflurane in room air and images were generated using a DICOM reader (OsiriX; University of Geneva, Switzerland). MRI scans of the brain were performed using respiratory-gated T2- weighted rapid acquisition of refocused echoes (RARE) MRI images acquired on a 4.7-T small animal scanner (Pharmascan; Bruker, Billerica, MA, USA) with the following parameters: RARE factor: 10, echo time: 60 ms, repetition time: 6000 ms, Averages: 8, 192 × 192 × 24 image matrix with a voxel size of 0.130 × 0.130 × 0.7 mm).

Sex as a biological variable

To establish the proof of principle that PT/GBT lowers brain PbO2, we used wild-type male mice. For the *Ndufs4* KO mouse experiments, data from both sexes is combined as we found no differences between males and females, and no sex differences in disease phenotypes have been reported.

Statistics

Analyses were performed using GraphPad Prism software. We performed one-way ANOVA with Dunnett's tests for multiple comparisons to vehicle, T-tests for single comparisons of GBT440/PT2399 to vehicle, and Log-rank test for survival of drug vs. vehicle-treated mice. A P-value <0.05 was considered to indicate statistical significance. Data are reported as mean ± SD.

Study approval

The Massachusetts General Hospital Institutional Animal Care and Use Committee approved all animal work in this manuscript.

Data availability

Supplementary XLS file includes data points underlying graphed data in Figure 1.

AUTHOR CONTRIBUTIONS

H.W., M.M., E.M., P.L., G.W. performed experiments, F.I. and V.K.M. supervised the research. H.W., M.M. and V.K.M. wrote the manuscript.

SUPPLEMENTARY REFERENCES

1. Kruse SE, Watt WC, Marcinek DJ, Kapur RP, Schenkman KA, and Palmiter RD. Mice with mitochondrial complex I deficiency develop a fatal encephalomyopathy. Cell Metabolism. 2008;7(4):312 20.

2. Quintana A, Kruse SE, Kapur RP, Sanz E, and Palmiter RD. Complex I deficiency due to loss of Ndufs4 in the brain results in progressive encephalopathy resembling Leigh syndrome. Proceedings of the National Academy of Sciences. 2010;107(24):10996 1001.

3. Feng Z, Zou X, Chen Y, Wang H, Duan Y, and Bruick RK. Modulation of HIF-2alpha PAS-B domain contributes to physiological responses. Proc Natl Acad Sci U S A. 2018;115(52):13240-5.
